# Zika virus cleaves GSDMD to disseminate prognosticable and controllable oncolysis in a human glioblastoma cell model

**DOI:** 10.1016/j.omto.2022.12.008

**Published:** 2023-01-02

**Authors:** Yu-Ting Kao, Hsin-I Wang, Chi-Ting Shie, Chiou-Feng Lin, Michael M.C. Lai, Chia-Yi Yu

**Affiliations:** 1 National Institute of Infectious Diseases and Vaccinology, National Health Research Institutes, Miaoli 350, Taiwan; 2 Department of Microbiology and Immunology, Taipei Medical University, Taipei 110, Taiwan; 3 Research Center for Emerging Viruses, China Medical University Hospital, Taichung 404, Taiwan; 4 Institute of Molecular Biology, Academia Sinica, Taipei 115, Taiwan; 5 Department of Microbiology and Immunology, National Cheng Kung University, Tainan 701, Taiwan

**Keywords:** Zika virus, GSDMD, oncolysis, glioblastoma cell, pyroptosis, protease

## Abstract

Glioblastoma (GBM) is the most common aggressive malignant brain cancer and is chemo- and radioresistant, with poor therapeutic outcomes. The “double-edged sword” of virus-induced cell death could be a potential solution if the oncolytic virus specifically kills cancer cells but spares normal ones. Zika virus (ZIKV) has been defined as a prospective oncolytic virus by selectively targeting GBM cells, but unclear understanding of how ZIKV kills GBM and the consequences hinders its application. Here, we found that the cellular gasdermin D (GSDMD) is required for the efficient death of a human GBM cell line caused by ZIKV infection. The ZIKV protease specifically cleaves human GSDMD to activate caspase-independent pyroptosis, harming both viral protease-harboring and naive neighboring cells. Analyzing human GSDMD variants showed that most people were susceptible to ZIKV-induced cytotoxicity, except for those with variants that resisted ZIKV cleavage or were defective in oligomerizing the N terminus GSDMD cleavage product. Consistently, ZIKV-induced secretion of the pro-inflammatory cytokine interleukin-1β and cytolytic activity were both stopped by a small-molecule inhibitor targeting GSDMD oligomerization. Thus, potential ZIKV oncolytic therapy for GBM would depend on the patient’s GSDMD genetic background and could be abolished by GSDMD inhibitors if required.

## Graphical abstract

**Figure:**
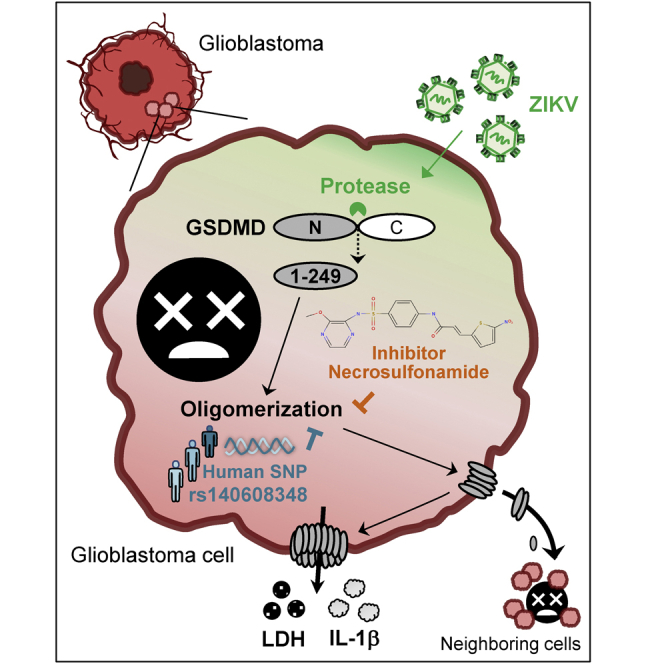

## Teaser

Kao et al. revealed that the Zika virus oncolytic activity against glioblastoma relies on viral protease-mediated human species-specific GSDMD cleavage. Releasing the cleaved GSDMD product further disseminates oncocidal effects to nearby uninfected cells. Thus, virus-mediated oncolytic outcomes could be predicted and manipulated by targeting GSDMD activation.

## Introduction

Cancer is one of the leading causes of death in the world. Among brain tumors, glioblastoma (GBM) was classified as a grade IV glioma by the World Health Organization because of its malignancy.^1^ This aggressive tumor sustains proliferation, immune escape, and drug resistance because GBM stem cells are multipotent, self-renewing, and apoptosis resistant.^2^^,^^3^ Despite the availability of surgery followed by radiotherapy and chemotherapy for treatment, the effectiveness of current treatments is poor, and recurrence is common.^4^ New strategies that specifically kill GBM cells are needed.

An oncolytic virus could be desirable for cancer therapy if the virus selectively kills the infected cancer cells with no harm to normal cells.^5^^,^^6^ Regardless of DNA or RNA as the genome, several viruses have been proposed as oncolytic viruses, including herpes simplex virus 1, vaccinia virus, reovirus, adenovirus, parvovirus, poliovirus, and Newcastle disease virus.^7^^,^^8^^,^^9^ Recently, Zika virus (ZIKV), which causes microcephaly in the fetus,^10^^,^^11^ was defined as an oncolytic virus because it preferentially infects and kills GBM stem cells^12^^,^^13^ but seems to not harm adult neurons.^14^^,^^15^ Although both adult and pediatric brain tumor cells could be killed by ZIKV,^12^ adult GBMs were preferentially studied to illustrate the oncolytic mechanisms, e.g., the requirement of T cells to increase the efficacy.^16^^,^^17^ The ZIKV neurotropism may result from the specific expression of SOX2 and α_v_β_5_ on GBM stem cells.^18^ The expression of the ZIKV receptor tyrosine kinase AXL renders GBM cells highly permissive to ZIKV and thus the killing effects; however, expression of the viral receptor is the prerequisite for virus infection but does not guarantee the killing.^19^ How ZIKV kills GBM cells after infection needs in-depth examination to assess the pros and cons of precision virotherapy.

Among various types of virus-induced cell death, pyroptosis was recently defined as gasdermin family protein-mediated cell death accompanying inflammatory responses mechanistically distinct from apoptosis.^20^^,^^21^^,^^22^ Among the six human gasdermin family members, the best characterized are gasdermin D (GSDMD) and E (GSDME), mainly activated by caspase-mediated cleavage.^23^ The proteolytic cleavage releases the N-terminal pore-forming domain to oligomerize and form pores on the cell membrane, thus leading to cell swelling, membrane rupture, and eventually cell lysis.^24^^,^^25^ The pores also facilitate the release of pro-inflammatory cytokines interleukin-1β (IL-1β)^26^ and IL-18^27^ and the cytokine mediator high-mobility group box 1,^28^ thus contributing to inflammatory pyroptosis.

Here, we found ZIKV induces cytolysis by caspase-independent pyroptosis besides apoptosis. The ZIKV protease specifically cleaved GSDMD for pyroptosis to kill infected and nearby uninfected cells. The GSDMD variants and small-molecule inhibitors could dominate ZIKV-triggered cytotoxicity outcomes. Thus, ZIKV as an oncolytic virotherapy against GBM cells could be genetically prognostic and pharmacologically regulated.

## Results

### Caspase-independent pyroptosis governs ZIKV-induced human GBM cell death

**Figure 1:**
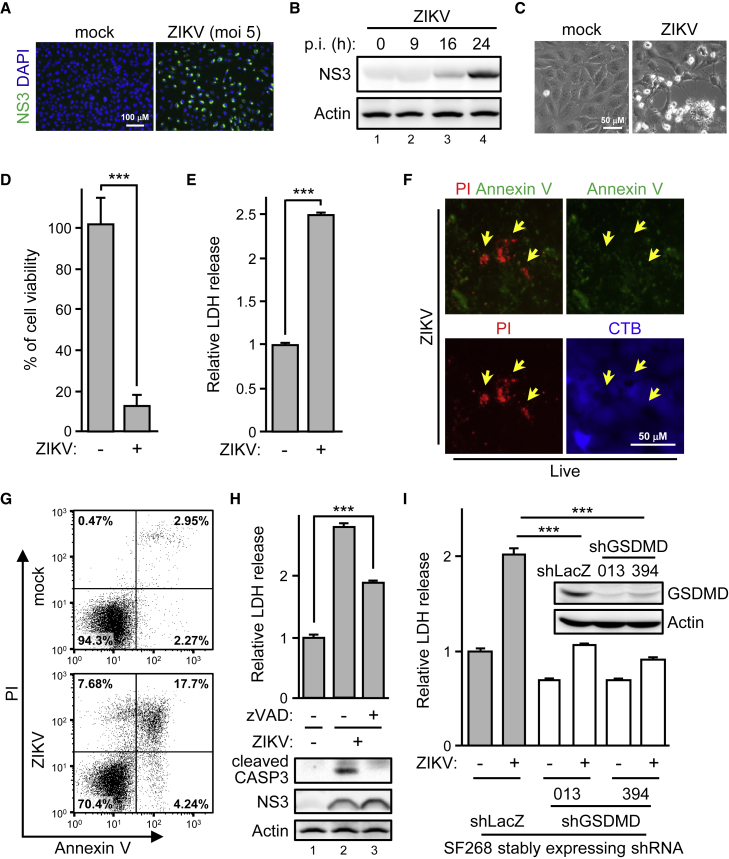
ZIKV-induced caspase-independent cell lysis in human glioblastoma cells (A–E) SF268 cells were infected with ZIKV. Cells were stained with anti-NS3, and nuclei were counterstained with DAPI (A). ZIKV protein level examined by Western blot analysis (B). p.i., post-infection. The morphology of the cytopathic effect of ZIKV observed by phase-contrast imaging (C). Cell viability measured by trypan blue exclusion assay (D). The supernatant was analyzed for lactate dehydrogenase (LDH) release (E). ZIKV-infected SF268 cells were live stained with propidium iodide (PI; red), annexin V (green), and control counterstain CellTracker Blue (CTB; blue) (F). Cells were photographed by fluorescence microscopy. Yellow arrows, PI-positive but annexin V-negative cells. SF268 cells were live stained with PI and annexin V and then analyzed by flow cytometry (G). SF268 cells were infected with ZIKV in the absence (−) or presence (+) of zVAD (50 μM) (H). Released LDH in the supernatant (top panel) and the expression of indicated proteins in the cell lysates (bottom panel). CASP3, caspase-3. SF268 cells stably expressing shRNA targeting control LacZ and human GSDMD confirmed by western blot analysis (I). The shLacZ- or shGSDMD-SF268 cells were infected with ZIKV for LDH release assay. #013 and #394 represent two different shRNAs targeting GSDMD. Data are mean ± SD, n = 3 per group. ∗∗∗p < 0.001.

To evaluate the ZIKV oncolytic potential, we established a ZIKV infection model in a cell culture system by using the human GBM cell line SF268. ZIKV infection was confirmed by immunostaining for ZIKV protein (Figures 1A and 1B). Phase-contrast imaging showed that ZIKV induced cytotoxicity (Figure 1C), which was further quantified by measuring cell viability (Figure 1D) and lactate dehydrogenase (LDH) released in the culture supernatant (Figure 1E). Propidium iodide (PI) staining revealed dead cells and annexin V staining ZIKV-induced apoptosis (Figure 1F). Because not all the dead cells (PI positive) were annexin V positive (Figure 1G), we wondered whether the ZIKV oncolytic activity could be non-apoptotic. This scenario was confirmed by the pan-caspase inhibitor zVAD partially blocking the ZIKV-induced cytotoxicity (Figure 1H, top panel) when ZIKV-activated caspase-3 was mainly blocked (Figure 1H, bottom panel). To test whether pyroptosis was responsible for the caspase-independent oncolysis upon ZIKV infection, we knocked down the expression of endogenous GSDMD in SF268 cells. ZIKV-triggered LDH release in shLacZ control cells was significantly reduced by silencing GSDMD with the two independent short hairpin RNAs (shRNAs) (#013 and #394) (Figure 1I). Thus, ZIKV infection triggered caspase- and GSDMD-mediated cytotoxicity in human GBM SF268 cells.

### GSDMD is required for ZIKV-induced cytotoxicity

**Figure 2:**
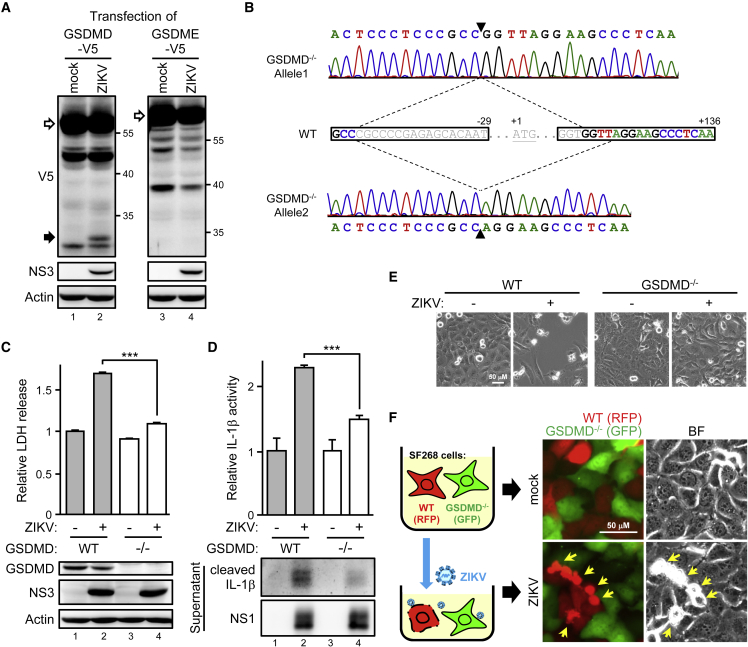
GSDMD is responsible for ZIKV-induced cytotoxicity in human glioblastoma cells (A) A549 cells with or without ZIKV infection were transfected with V5-tagged GSDMD or GSDME. Cell lysates were examined by western blot analysis. White arrows, full-length GSDMD or GSDME; black arrow, cleaved product. (B) GSDMD genomic DNA sequencing of SF268 wild-type (WT) and knockout (*GSDMD*^−/−^) cells. The single guide RNA sequence designed for CRISPR-Cas9 genome editing is marked in a black frame. (C–E) WT and *GSDMD*^−/−^ SF268 cells were infected with ZIKV for western blot analysis (C, bottom panel), LDH release (C, top panel), IL-1β secretion (D), and phase-contrast imaging (E). Data are mean ± SD (n = 3 per group). ∗∗∗p < 0.001. (F) WT (stably RFP-labeled) and *GSDMD*^−/−^ (stably GFP-labeled) SF268 cells were cocultured overnight and synchronize infected with ZIKV for another 24 h, then were examined by fluorescent and phase-contrast microscopy. Yellow arrows, damaged cells.

To understand how ZIKV induces pyroptosis in human cells, we first used an infection-transfection A549 cell model to check cleavage of the pyroptosis marker gasdermin family protein. A cleaved product of GSDMD, but not GSDME, was readily detected in ZIKV-infected cells (Figure 2A). To clarify the roles of GSDMD in ZIKV infection without the effects of incomplete GSDMD knockdown (Figure 1I), we knocked out endogenous GSDMD in SF268 cells by using the CRISPR-Cas9 system. GSDMD knockout (*GSDMD*^−/−^) was verified by sequencing the genomic DNA of SF268-*GSDMD*^−/−^ cells (Figure 2B). Consistent with the GSDMD knockdown cells, ZIKV-induced cytotoxicity was greatly reduced by GSDMD knockout (Figure 2C). In line with the GSDMD-mediated pyroptosis accompanying inflammatory cytokine IL-1β production, the bioactivity and quantity of ZIKV-induced IL-1β was greatly reduced by GSDMD ablation (Figure 2D). Phase-contrast imaging revealed ZIKV-induced killing of wild-type (WT) GBM cells, with infected *GSDMD*^−/−^ cells remaining intact (Figure 2E). We used ZIKV to infect a coculture pool of RFP-labeled WT and GFP-labeled *GSDMD*^−/−^ cells to confirm the observation. The synchronized ZIKV infection revealed that WT, rather than *GSDMD*^−/−^, cells were more sensitive to the ZIKV killing effects (Figure 2F). Thus, GSDMD positively contributed to the ZIKV-induced cell death and pro-inflammatory cytokine secretion in human GBM SF268 cells.

### ZIKV protease is sufficient to trigger GSDMD-mediated cell death

**Figure 3:**
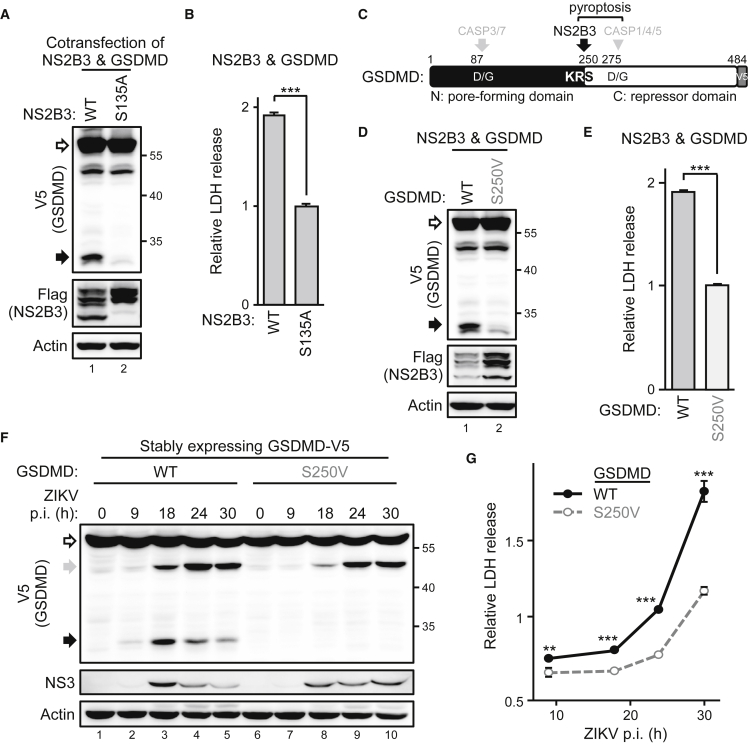
ZIKV protease governs cytolytic activity by cleaving GSDMD (A and B) 293T/17 cells were cotransfected with ZIKV protease NS2B3 (WT; S135A, the protease-dead mutant) and GSDMD. The protein levels were examined by western blot analysis (A), and the supernatant was analyzed for LDH release (B). (C) Schematic illustration of human GSDMD with the functional domains and protease cleavage sites. (D and E) 293T/17 cells were transfected with ZIKV protease and each indicated GSDMD and examined by western blot analysis (D) and LDH release assay (E). (F and G) Vero cells stably expressing each indicated GSDMD-V5 were infected with ZIKV for the indicated time and examined by western blot analysis (F) and LDH release assay (G). White arrows, full length; black and gray arrows, cleaved products. Data are mean ± SD (n = 3 per group). ∗∗p < 0.01, and ∗∗∗p < 0.001.

The NS2B3 should be the only exogenous protease introduced by ZIKV infection despite the various host proteases that could be activated. Activating canonical pyroptosis depends on the cellular caspases,^29^ so we wondered whether ZIKV triggers caspase-independent pyroptosis by the viral protease. Cotransfection of ZIKV protease NS2B3 and GSDMD showed that expressing the viral protease alone, but not its protease-dead mutant (S135A), was sufficient to cleave GSDMD (Figure 3A) and trigger cell death (Figure 3B). The cleavage seemed species specific because missing the corresponding cleavage site makes the murine GSDMD resistant to ZIKV protease (Figure S1), indicating species concerns of animal models used in ZIKV therapy studies. According to the cleaved GSDMD product molecular weight, and putative viral cleavage site motifs (a short side chain following two basic residues)^30^ analysis, ZIKV protease seemed to specifically cleave GSDMD at the KR↓S^250^ (Figure 3C). This hypothesis was further confirmed by the cotransfection of ZIKV protease with the GSDMD(S250V) mutant. ZIKV protease cleaved the GSDMD(WT), but the GSDMD(S250V) mutant was resistant to the cleavage (Figure 3D). Hence, ZIKV protease-triggered cytotoxicity was greatly attenuated by the S250V mutant (Figure 3E). The cellular caspase- (gray arrows and triangle) and ZIKV protease-mediated GSDMD cleavage products (black arrows) were further investigated by treating the ZIKV-infected cells harboring the GSDMDs of interest with zVAD or not (Figure S2). To understand the timeline of GSDMD-mediated cell death after ZIKV infection, we checked the GSDMD cleavage events in cells stably expressing the V5-tagged GSDMDs. ZIKV protease-mediated GSDMD cleavage was detected as early as 9 h (Figure 3F, lane 2, black arrow), but the caspase-mediated GSDMD cleavage was observed later, at 18 h (Figure 3F, lane 3, gray arrow). Of note, ZIKV protease-resistant GSDMD(S250V) slightly reduced the caspase-mediated cleavage (Figure 3F, lane 3 versus 8, gray arrow), which suggests ZIKV protease-intiated, rather than caspase-initiated, pyroptosis in ZIKV-infected SF268 cells. At all events, cells with GSDMD(S250V) showed more resistance to ZIKV-induced cytotoxicity than did GSDMD(WT) cells (Figure 3G) after ZIKV infection. In sum, ZIKV protease alone was sufficient to induce cell death by cleaving GSDMD at the KR↓S^250^ site.

### Cleaved GSDMD disseminates damage over adjacent cells

**Figure 4:**
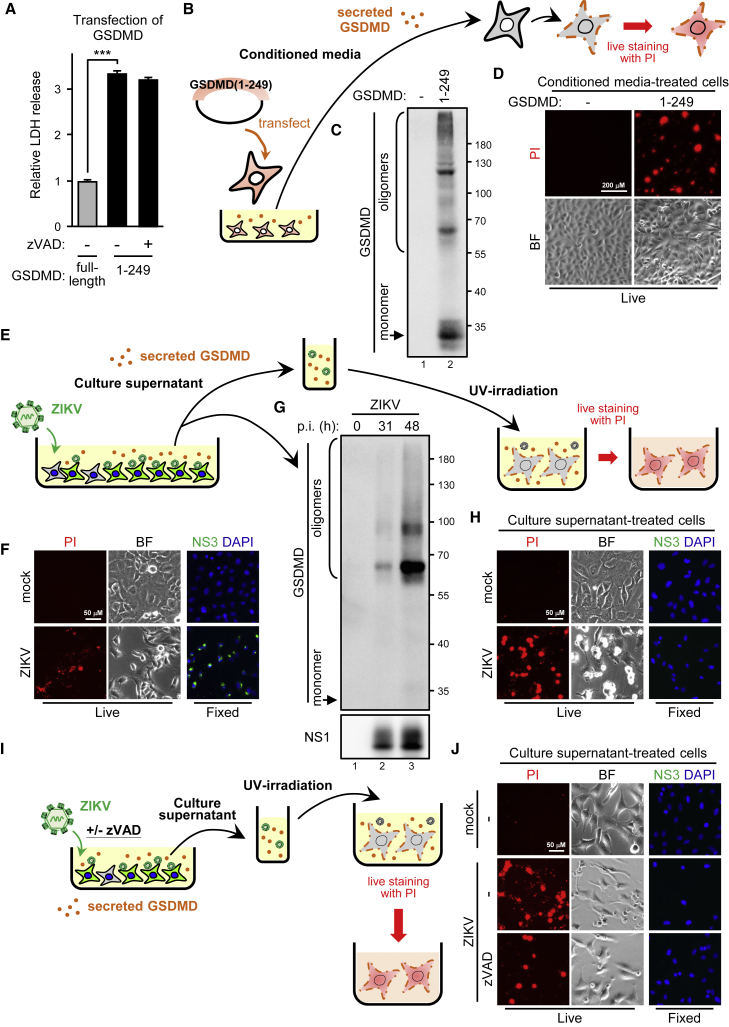
Transferring the cleaved GSDMD product produces cell death in neighboring cells (A) 293T/17 cells were transfected with GSDMD full-length or GSDMD cleavage product mimic 1–249 in the absence (−) or presence (+) of zVAD (50 μM). Released LDH was analyzed. Data are mean ± SD (n = 3 per group). ∗∗∗p < 0.001. (B–D) Experimental design to study the effects of cleaved GSDMD product on neighboring cells (B). 293T/17 cells were transfected with GSDMD(1–249), and the supernatant was harvested and subjected to western blot analysis of secreted GSDMD (C). The supernatant was then used as the conditioned media to treat naive Vero cells. Red, PI live staining; BF, bright field (D). (E–H) Experimental design of study of the effect of ZIKV-activated GSDMD on neighboring cells (E). ZIKV-infected SF268 cells were live stained with PI (red) or fixed stained with anti-NS3 antibody (green) and DAPI (blue) (F). The culture supernatant of ZIKV-infected SF268 cells was examined by western blot analysis (G). The culture supernatant from mock- or ZIKV-infected SF268 cells was UV inactivated and used as the conditioned media incubating the naive SF268 cells. Cells were live stained with PI to check viability or fixed stained with DAPI and anti-NS3 antibody to confirm viruses were UV inactivated (H). PI, red; NS3, green; DAPI, blue. (I and J) The experiment carried out in the presence or absence of zVAD (I). Naive SF268 cells were incubated with the UV-inactivated conditioned media from mock- or ZIKV-infected SF268 cells for 24 h. Cells were live stained with PI or fixed stained with anti-NS3 antibody and DAPI (J).

ZIKV elicited an extremely high level of cell damage in SF268 cells expressing GSDMD(WT) but not GSDMD(S250V) (Figure 3G), indicating that the viral protease-mediated GSDMD cleavage might amplify cell demolition to cause massive cell death. Thus, we transfected the cytotoxic GSDMD cleavage product mimic GSDMD(1–249) into cells to test this hypothesis. As expected, an LDH release assay showed that the N-terminal GSDMD cleavage product caused massive death of the transfectant cells regardless of caspase activities (Figure 4A). Analyzing the culture supernatant of GSDMD(1–249) transfectants (Figure 4B) showed that the 1–249 mimics were also released and formed oligomers, which may permeabilize the cell membrane, thus leading to rupture (Figure 4C). Hence, we cultured fresh Vero cells with conditioned media to determine whether the secreted GSDMD(1–249) harms naive cells. Live staining assay showed that conditioned media harboring GSDMD(1–249) were sufficient to permeabilize the naive Vero cell membrane for PI staining (Figure 4D). We infected SF268 cells with ZIKV to check whether the GSDMD(1–249)-mediated remote-killing phenomenon was reflected in natural ZIKV infection of GBM cells (Figure 4E). At 31 h post-infection, when the ZIKV-infected cells were permissive to PI staining (Figure 4F), secreted GSDMD was also detected in the culture supernatant (Figure 4G). The conditioned culture media was then UV irradiated to inactivate ZIKV and used to treat naive SF268 cells. In the absence of infectious ZIKV, the conditioned media remained able to damage the naive SF268 cell membrane (Figure 4H). Consistently, live staining of the cell surface ZIKV NS1 and PI thus showed that uninfected cells (NS1 negative) could die (PI positive) from being exposed to a cytotoxic environment created by the infected cells (Figure S3). The pan-caspase inhibitor zVAD did not block the ZIKV-induced GSDMD-mediated cell injury (Figures 4I and 4J), which suggests that ZIKV infection harmed the infected human GBM cells and disseminated GSDMD-mediated injury to nearby uninfected cells.

### GSDMD single-nucleotide polymorphisms (SNPs) affect ZIKV-induced cytotoxicity

**Figure 5:**
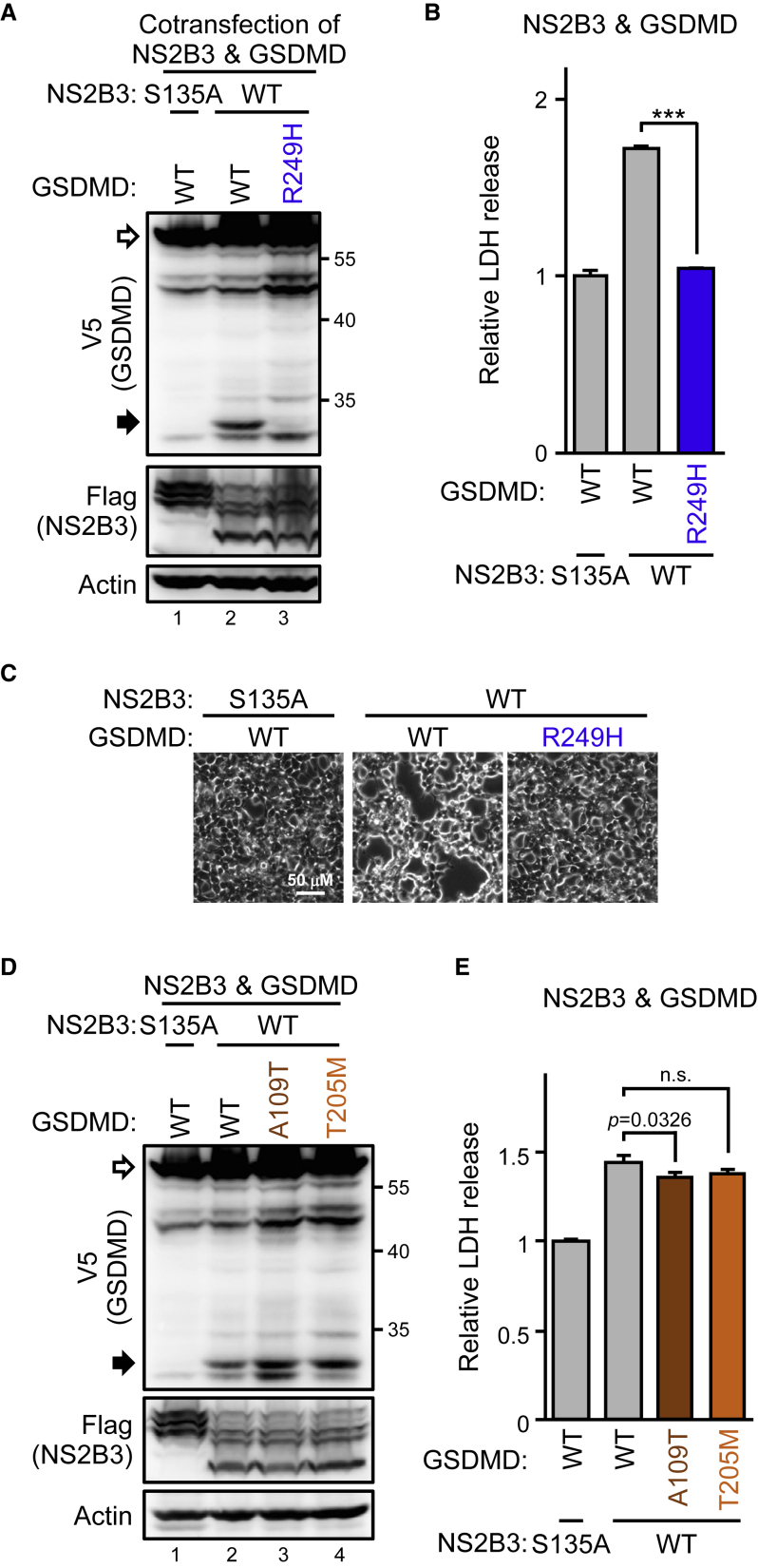
GSDMD variants with high frequency in Taiwan remain sensitive to ZIKV protease cleavage 293T/17 cells were cotransfected with the ZIKV protease and the indicated GSDMDs. Cell lysates were examined by western blot analysis (A and D), and the supernatant was collected for LDH assay (B and E). Phase-contrast photography is shown in (C). White arrows, full-length GSDMD-V5; black arrows, cleaved GSDMDs. Data are mean ± SD (n = 3 per group). ∗∗∗p < 0.001, and n.s., not significant.

ZIKV activates pyroptosis by viral protease, specifically cleaving GSDMD at KR↓S^250^, which suggests that oncolytic ZIKV viral therapy would depend on the GSDMD vulnerability to ZIKV protease. We replaced the S residue of human GSDMD with the corresponding residue V found in murine and restored the SF268-*GSDMD*^−/−^ cells with either the WT or S250V mutant for ZIKV infection. ZIKV-induced LDH release (Figure S4A), IL-1β production (Figure S4B), and disseminated GSDMD-mediated injury to neighboring uninfected cells (Figures S4C and S4D) were all attenuated with the S250V mutant. Thus, blocking caspase-1-mediated GSDMD activation (Figure S4E, lane 4, gray triangle) could reduce ZIKV-induced cytotoxicity in the cells with the S250V mutant to the level comparable to the absence of endogenous GSDMD (Figure S4F). We next checked whether the natural SNP of human GSDMD affects ZIKV-induced cell killing. As expected, the GSDMD R249H variant (SNP rs138749323) resisted ZIKV protease-mediated cleavage (Figure 5A) and thus did not trigger cell death (Figures 5B and 5C).

We next checked other natural SNPs from two databases to investigate the *GSDMD* genetic background for potential ZIKV oncotherapy responders. Overall, 5,616 *GSDMD* SNPs were identified in the global dbSNP database, and 175 GSDMD variants were in the local Taiwan BioBank.^31^ We narrowed down the SNPs of interest to the coding region (593 in dbSNP, 12 in Taiwan BioBank), which resulted in missense variants (379 in dbSNP, 7 in Taiwan BioBank) (Figures S5A and S5B). Because activated GSDMD forms pores to permeabilize the cell membrane, we focused on the two missense variants, A109T (SNP rs200806004) and T205M (SNP rs149736517), that reside in the pore-forming domain (Figures S5B and S5C). Cotransfection of the GSDMD variants with ZIKV protease showed that both A109T and T205M variants remained susceptible to ZIKV protease (Figure 5D) and capable of mediating cell death (Figure 5E) after cleavage. Thus, we did not find the *GSDMD* genetic background of possible ZIKV oncotherapy non-responders in Taiwanese people by this strategy.

### The variant and inhibitor disturbing GSDMD oligomerization attenuates ZIKV-induced cell lysis

**Figure 6:**
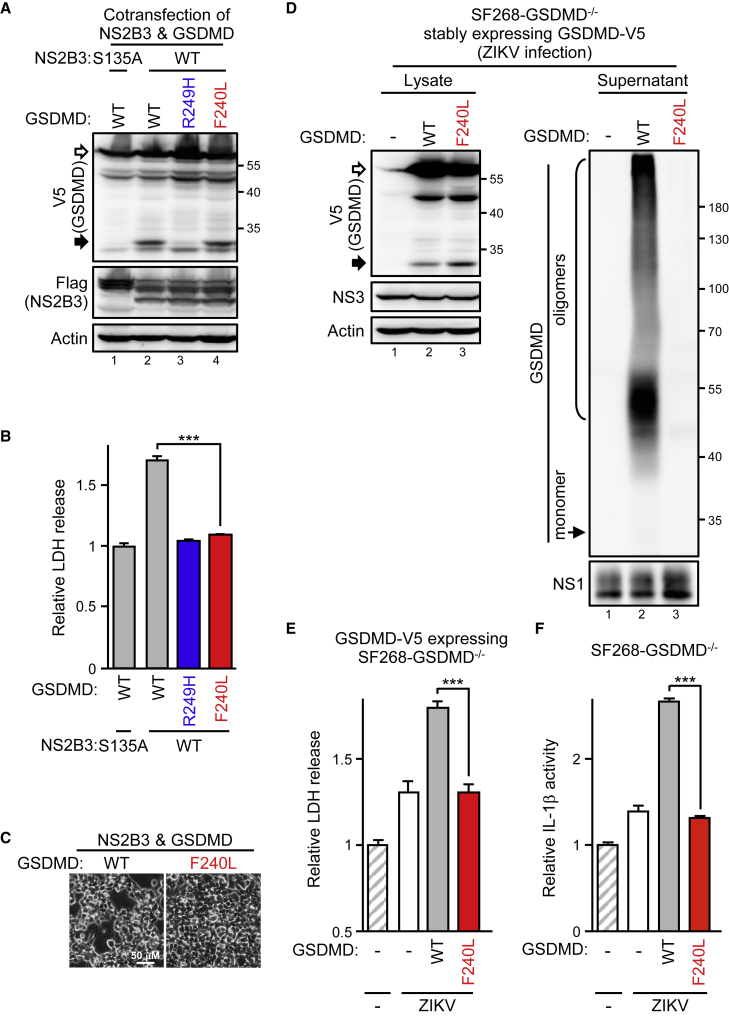
GSDMD oligomerization-deficient variant decreases ZIKV-induced cytotoxicity (A–C) 293T/17 cells were cotransfected with the ZIKV protease and the GSDMD variants as indicated. Cell lysates were examined by western blot analysis (A), and the supernatant was collected for LDH assay (B). Phase-contrast photography is shown in (C). (D–F) SF268-*GSDMD*^−/−^ cells stably restored with each V5-tagged GSDMD were infected with ZIKV as indicated. Cell lysates and the culture supernatant were examined by western blot analysis (D). The supernatant was also measured for LDH release (E) and IL-1β secretion (F). White arrows, full-length GSDMD-V5; black arrows, ZIKV protease-cleaved GSDMD products. Data are mean ± SD (n = 3 per group). ∗∗∗p < 0.001.

Although most human GSDMDs seemed capable of mediating ZIKV protease-triggered oncolysis, the marginal cell death attenuation of the A109T variant drew our attention. The A109T GSDMD variant resides in the pore-forming domain, which suggests that a variant with defects in GSDMD pore formation might prevent ZIKV-triggered pyroptosis. Therefore, we investigated the F240L variant (SNP rs140608348; Figure S5D) that aborts GSDMD oligomerization.^32^ Because the F240L variant did not affect the KR↓S^250^ cleavage site, the ZIKV protease remained able to cleave GSDMD(F240L) and the GSDMD(WT) in a cotransfection assay (Figure 6A). However, cleaving F240L did not induce LDH release (Figure 6B) or cell damage (Figure 6C) as did cleaving GSDMD(WT). Therefore, we restored the SF268-*GSDMD*^−/−^ cells with the WT or F240L variant for ZIKV infection. ZIKV protein expression seemed equivalent among *GSDMD*^−/−^ cells restored with the GSDMD(WT) or GSDMD(F240L) variant: both the WT and F240L remained sensitive to ZIKV cleavage, but F240L did not form oligomers and secrete after the cleavage (Figure 6D). Consistently, ZIKV-induced LDH release (Figure 6E) and IL-1β production (Figure 6F) were greatly attenuated with the F240L variant as for without endogenous GSDMD. This finding was also supported by the GSDMD cleavage product mimic with F240L losing its cytotoxicity (Figure S6). Thus, targeting GSDMD oligomerization could abolish ZIKV-induced cytotoxicity and the inflammatory response.

**Figure 7:**
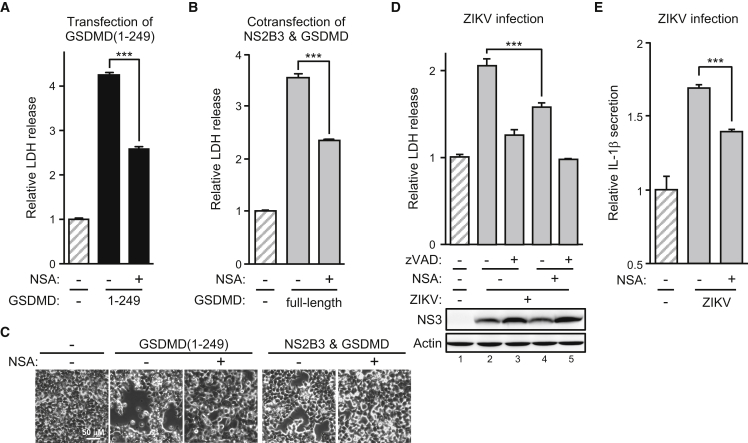
GSDMD oligomerization inhibitor interferes with ZIKV-induced oncolysis (A) 293T/17 cells were transfected with GSDMD(1–249) in the absence (−) or presence (+) of necrosulfonamide (NSA; 50 μM). Released LDH was analyzed. (B) 293T/17 cells were cotransfected with full-length GSDMD and NS2B3 in the absence (−) or presence (+) of NSA (50 μM). Released LDH was analyzed. (C) 293T/17 cells were transfected with GSDMD(1–249) or cotransfected with full-length GSDMD and NS2B3 in the absence (−) or presence (+) of NSA (50 μM). Phase-contrast photography is shown. (D and E) SF268 cells were infected with ZIKV in the absence (−) or presence (+) of NSA (10 μM) or zVAD (50 μM). Released LDH (top panel) was analyzed and cell lysates (bottom panel) were analyzed for NS3 protein level (D), and IL-1β secretion was determined (E). Data are mean ± SD (n = 3 per group). ∗∗∗p < 0.001.

Because ZIKV protease-triggered pyroptosis depended on the cleaved GSDMD product pore-forming capability, we sought some small molecules targeting this step for better pharmacological control of ZIKV-induced oncolysis if required. We tested the killing effects under treatment with necrosulfonamide (NSA), a small molecule directly interacting with GSDMD to prevent its oligomerization.^33^ NSA successfully protected SF268 cells against death triggered by GSDMD(1–249) (Figure 7A) or ZIKV protease-cleaved GSDMD (Figures 7B and 7C). NSA alone was sufficient to reduce ZIKV-induced cell death (Figure 7D) and IL-1β secretion (Figure 7E). We tested another inhibitor, punicalagin (PUN), which reversibly blocks plasma membrane permeabilization,^34^ for protection against ZIKV-induced cell death. PUN successfully attenuated cell death triggered by transfecting GSDMD(1–249) (Figure S7A), cotransfecting full-length GSDMD with ZIKV protease (Figure S7B), or ZIKV infection (Figure S7C). However, PUN did not rescue ZIKV-induced IL-1β production (Figure S7D), probably because of the significant but marginal protection effect of PUN. Together, the results indicate that ZIKV protease-triggered, GSDMD-mediated oncolysis depends on the host *GSDMD* genetic background and could be pharmacochemically aborted if needed.

## Discussion

Virus-induced cell death could be a “double-edged sword” for the infected host: killing the healthy tissue cells is pathogenic, but eliminating the malignant tumor cells is therapeutic. ZIKV seems preferentially harmful to the neurons of fetuses and children^35^ but seldomly adults, so pathogenic ZIKV infection could be a potential oncolytic therapy against adult GBM.^36^^,^^37^ However, a safe and aspirational viral therapy requires investigation of the mechanism of how, beyond the phenotypic observations, the virus causes specific cell death. Here, we showed that ZIKV could be an oncolytic virus by killing human GBM via viral protease-mediated GSDMD activation. The species-specific GSDMD cleavage also suggested that a humanized animal or human *GSDMD* transgenic mouse rather than a simple murine GBM model would provide convincing experimental conclusions of preclinical ZIKV oncotherapy.

GSDMD is a novel biomarker for evaluating the cancer prognosis because of the high protein expression level in glioma and its association with significant survival of GBM patients.^38^ GSDMD-mediated pyroptosis accompanies the release of the pro-inflammatory cytokine IL-1β,^26^^,^^39^ which supports the tumor-specific T helper 1 cell-mediated immune response against cancer.^40^ The conventional pyroptosis release of mature IL-1β is by caspase-1- or caspase-11/4/5-mediated GSDMD cleavage that releases the GSDMD-N perforating the plasma membrane.^29^^,^^41^ Because the process can be negatively regulated by caspase-3-mediated cleavage of GSDMD-N,^42^ the ZIKV protease-activated pyroptosis might be manipulated by infection-elicited caspases. Whether the ZIKV-induced caspase profiles or specific caspase inhibitors affect the therapeutic outcomes of ZIKV tumor therapy awaits further investigation.

Virulence factors contributing to ZIKV-induced cell death need to be identified to provide the foundations of applying ZIKV. Overexpressing ZIKV E protein alone is sufficient to suppress cell proliferation and induce caspase-mediated apoptosis.^43^ A 10-nt deletion in the 3′ untranslated region of the ZIKV genome produces a live-attenuated ZIKV both *in vitro* and *in vivo* and has been proposed as a vaccine candidate against ZIKV^44^ and as virotherapy against malignant GBM.^45^ Therefore, to eliminate uncertainty about other ZIKV components, nanoparticles or single-round infectious particles harboring the ZIKV protease or GSDMD-N should be safer than the live virus for cancer therapy.

Among various proteins and cytokines secreted after infection, GSDMD-N release upon ZIKV infection interested us. The released GSDMD-N may harm the uninfected tumor cells to expand the therapeutic effects or normal tissue to cause side effects. Considering that GBM is a solid, not a liquid, tumor, with local GSDMD-N secretion and ZIKV replicating capability, a small dose of ZIKV would be sufficient for the therapy. The extracellular GSDMD-N may also benefit patients with brain cancer who are susceptible to bacterial infection because GSDMD is an antibacterial peptide.^46^ Of note, the enterovirus protease 3C stops pyroptosis by further cleaving GSDMD-N,^47^ so ZIKV oncotherapy would be complicated when coinfecting microbes capable of antagonizing GSDMD activation.

To date, no ZIKV-based clinical trial has been conducted for oncology therapies. Pros and cons coexist and complicate the virotherapy. A suitable animal model for ZIKV therapy against human GBM remains for development, but we demonstrated that ZIKV protease-activated GSDMD is involved in the management and prognosis of ZIKV therapy. A GSDMD genetic background could be used to screen positive responders for the virotherapy. In an out-of-control ZIKV infection, ZIKV protease should be the target to stop the treatment. Nevertheless, the small molecules regulating GSDMD activation might serve to abort the virotherapy independent of ZIKV protease activity. Until we obtain a better understanding of the mechanisms underlying ZIKV-induced cell lysis, our study provides a reference base for preclinical evaluation for predicting treatment outcomes and the possible impact of therapeutic effects combined with drugs targeting GSDMD oligomerization or caspase activity.

## Materials and methods

### Inhibitors

Caspase inhibitor I (zVAD) (627610) and NSA (480073) were from Merck (Kenilworth, NJ, USA).

### Plasmids

The cDNA of human GSDMD or GSDME was amplified from total cDNA from A549 cells by PCR with the primers 5′-GCGTCGACGGGTCGGCCTTTGAGCG-3′ and 5′- GCTCTAGACCGTGGGGCTCCTGGCTCAGT-3′ for GSDMD and 5′- GCGTCGACTTTGCCAAAGCAACCAGGAAT-3′ and 5′- GCTCTAGACCTGAATGTTCTCTGCCTAAAGCACAG-3′ for GSDME. The GSDMD mutants were obtained by single-primer PCR mutagenesis^48^ with GSDMD-V5 used as a template and the following primers: 5′-GCAAAGATCGCAGGCGGCGCCACGGTGTCTGACAGCTCCAGC-3′ for GSDMD(A109T); 5′-GGCCATCTGAGCCAGAAGAAGATGGTCACCATCCCCTCAGGATCCACCCTCGCATTCCGGGT-3′ for GSDMD(T205M); 5′-CGGATAAGAAGCAGAGGACCCTGCAGCCACCCGCGACA-3′ for GSDMD(F240L); 5′-CCAGCCACCCGCGACCGGTCACAAGCATTCCACGAGCGAAGGCG-3′ for GSDMD(R249H); and 5′-CGACAGGCCACAAGCGTGTCACGAGCGAAGGCGC-3′ for GSDMD(S250V). Truncated GSDMD(1–249) was cloned from GSDMD-V5 by PCR with the 5′-CCCAAGCTTACCATGGGGTCGGCCTTTGA-3′ and 5′-CTAGTCTAGACCACGCTTGTGGCCTGTCG-3′ primers. ZIKV NS2B3 was cloned by using the 5′-GTGTGGTGGAATTCGGCTGAAAATGAGCTGGCCCCCTAGCG-3′ and 5′-GACGCATGCGAATTCGGATCCTTCTTTTCCCAGCGGCAA-3′ primers. The S135A mutant of ZIKV NS2B3 was generated by single-primer mutagenesis^48^ with the primer 5′-GCTGGATTACCCAGCAGGAACCGCGGGATCTCCAATCCTAGACAAGTGTG-3′.

### Virus and cell lines

ZIKV strain PRVABC59 (GenBank: KU501215) was from the Centers for Disease Control, Taiwan. The human GBM cell line SF268 (RRID: CVCL_1689) was from Dr. Wen-Chi Su (China Medical University, Taichung, Taiwan). Cells were cultured in DMEM (SH30022.02, HyClone, Logan, UT, USA) containing 10% fetal bovine serum (FBS). Mosquito cell line C6/36 (CRL-1660, ATCC) was grown in RPMI (SH30027.01, HyClone) containing 5% FBS. Human lung epithelial carcinoma A549 cells (CCL-185, ATCC) were cultured in F-12K (21127-022, Gibco, Eugene, OR, USA) containing 10% FBS. Endogenous GSDMD-undetected human 293T/17 cells (CRL-11268, ATCC) were grown in DMEM containing 10% FBS. African green monkey kidney Vero cells (CCL-81, ATCC) were cultured in MEM (SF30024.02, HyClone) containing 10% FBS. Stably knocked down cells were established by transduction of the lentiviral vector harboring shLacZ (TRCN0000072224) or shGSDMD (TRCN0000180013, the #013; TRCN0000179394, the #394) from RNAi Core Facility at Academia Sinica, Taiwan. ZIKV was propagated in the C6/36 cells. The virus titer was measured by plaque-forming assay using Vero cells as described.^49^ For ZIKV infection assays, cells were adsorbed with ZIKV for 2 h, and the unbound viruses were removed with fresh media. Except for the specified multiplicity of infection (MOI) labeled in the figure, all the others were done using an MOI of 20.

### GSDMD knockout SF268 cells

The all-in-one CRISPR vector pAll-Cas9.Ppuro (RNAi core facility, Academia Sinica, New Taipai, Taiwan) was digested with *Bsm*BI and ligated with annealed oligonucleotides (GTTGTGCTCTCGGGGCGGGC and GTGAGGGCTTCCTAACCACC) for specific sgRNAs targeting human GSDMD. SF268 cells were transfected with adequate CRISPR plasmid by Lipofectamine 3000 Reagent (Thermo Fisher Scientific, Eugene, OR, USA) and then selected with puromycin (0.5 μg/mL) for additional 3 days. Single-cell clone candidates were obtained by limiting dilution method. Genomic DNA from candidates was subject for PCR check. A WT (840 bps) or knockout (676 bps) PCR amplicon spanning the target site was tested using the following primers: 5′-TACCGTAGACAACAGGGAGAACACTG-3' and 5′- AGAGTCTGCCAGGTGTTAGGGTCC-3′.

### Immunofluorescence assay

SF268 cells were fixed with 4% paraformaldehyde in phosphate-buffered saline (PBS; BF203-5L, Protech Technology, Taipei, Taiwan) for 30 min. After blocking with skim milk in PBS, cells were incubated with primary antibodies against NS3 (AS3-274)^50^ diluted in skim milk in PBS overnight, then with Alexa Fluor 488-conjugated goat antimouse antibody (A21042, Invitrogen, Rockford, IL, USA) for 1 h at room temperature, followed by nuclear DAPI counterstaining (0.25 ng/mL; D1306, Thermo Fisher Scientific). Cells were photographed by fluorescence microscopy (Olympus IX73).

### Western blot analysis

Cells were lysed with radioimmunoprecipitation assay (RIPA) buffer (10 mM Tris [pH 7.5], 5 mM EDTA, 150 mM NaCl, 0.1% SDS, 1% Triton X-100, 1% sodium deoxycholate) including a cocktail of protease inhibitors (04693132001, Roche, Mannheim, Germany). Equivalent amounts of proteins were separated on SDS-PAGE and transferred to a nitrocellulose membrane (XR-IGE-10600003, Amersham, Darmstadt, Germany). Non-specific antibody binding sites were blocked with skim milk in PBS with 0.1% Tween 20 (PBST), then reacted with the indicated primary antibodies for caspase-3 (#9662), GSDMD (#96458 and #97558), V5 tag (#13202), cleaved IL-1β (#83186), and Myc tag (#2278) from Cell Signaling Technology (Beverly, MA, USA); antibodies for ZIKV NS3 (GTX133309), actin (GTX629630), and ZIKV NS1 (GTX133323) from GeneTex (Irvine, CA, USA); and anti-FLAG (F1804) from Sigma-Aldrich (St. Louis, MO, USA), and then incubated with corresponding secondary antibodies. Signals were detected by Immobilon Western Chemiluminescence HRP Substrate (WBKLS0500, Millipore, Darmstadt, Germany) or SuperSignal West Femto Maximum Sensitivity Substrate (34096, Thermo Fisher Scientific) using UVP (ChemiDoc-It Imaging system, Analytik, Jena, Germany) with VisionWorks LS v.8.20 software.

### Trypan blue exclusion assay

Cells were mixed with Gibco Trypan Blue Solution (15250-061, Thermo Fisher Scientific), and surviving cells were counted for cell viability.

### Cytotoxicity test

Cell death was evaluated by detecting LDH release by using the Cytotoxicity Detection Kit (11644793001, Roche).

### Detection of cell death

Cells were sampled and live stained with CellTracker Blue (C2110, Invitrogen) for 15 min, then washed with medium and stained with PI and annexin V for 10 min with the Annexin V-FITC Apoptosis Detection Kit (ab14085, Abcam, Cambridge, UK), followed by fluorescence microscopy (Olympus IX73) to obtain photographs. To quantify cell death, cells were detached and stained with PI and annexin V for 10 min before analyzing by flow cytometry (BD FACSCalibur).

### Measurement of bioactive IL-1β

HEK-Blue IL-1β cells (hkb-il1bv2, InvivoGen, San Diego, CA, USA) were seeded at 150 μL per 96-well plate overnight, then incubated with 50 μL UV-inactivated conditioned media for another day. UV inactivation was done by using the GS Gene Linker UV Chamber (Bio-Rad, Hercules, CA, USA) following the program setting of sterilization application. For the confirmation of virus inactivation in the experiments, a parallel experiment was done side by side stained with ZIKV viral protein to ensure no virus was alive. HEK-Blue IL-1β cells sense bioactive IL-1β in conditioned media and then activate nuclear factor κB (NF-κB)/AP-1, leading to the production of secreted embryonic alkaline phosphatase (SEAP). The cell culture supernatant was transferred to a flat-bottom 96-well plate and mixed with 150 μL/well QUANTI-Blue Solution, a SEAP detection medium (rep-qbs, InvivoGen). After incubation, the bioactive IL-1β was represented by measuring the SEAP levels by spectrophotometry at 650 nm.

### Trichloroacetic acid solution (TCA) precipitation

TCA precipitation helped to detect trace proteins in the culture supernatant.^51^ Briefly, the secreted GSDMD in the culture supernatant was incubated with 1 mM disuccinimidyl suberate (DSS) (#21655, Thermo Fisher Scientific) for 30 min. The reaction was quenched by 1 M Tris-HCl (pH 7.5) for 10 min, and the protein samples were precipitated using 20% TCA (T0699, Sigma-Aldrich) at a ratio of 1:1. After incubation on ice for 30 min, the mixture was then centrifuged at 4°C at 15,000 × *g* for 15 min. The pellet was washed three times with ice-cold acetone and centrifuged at 4°C at 15,000 × g for 10 min. The supernatant was removed, the pellet was air dried, and then the pellet was reconstituted in 1× SDS sample buffer for western blot analysis.

### Quantification and statistical analysis

Data are shown as mean ± SD. Two-tailed Student’s t test was used to compare differences between two groups. p <0.05 was considered statistically significant.

## Data Availability

National Center for Biotechnology Information (NCBI) 1000 Genomes Project data (https://www.ncbi.nlm.nih.gov/SNP) are available from the National Cancer Institute, NIH. Taiwan BioBank data (https://taiwanview.twbiobank.org.tw) are available from Biomedical Translation Research Center in Academia Sinica, Taiwan. All data supporting the findings of the study are included in the paper and supplemental information.

## Supplemental information

Document S1. Figures S1–S7 and supplemental materials and methods

Document S2. Article plus supplemental information
