# Supplementary material for: Zika virus cleaves GSDMD to disseminate prognosticable and controllable oncolysis in a human glioblastoma cell model
Source: Mol Ther Oncolytics. 2023 Jan 2;28:104–17. doi: 10.1016/j.omto.2022.12.008 (PMC9845690; doi:10.1016/j.omto.2022.12.008)
Supplement: Document S1. Figures S1–S7 and supplemental materials and methods [file mmc1.pdf]

OMTO, Volume *28*

Supplemental information

Zika virus cleaves GSDMD to disseminate prognosticable and controllable oncolysis in a human glioblastoma cell model

Yu-Ting Kao, Hsin-I Wang, Chi-Ting Shie, Chiou-Feng Lin, Michael M.C. Lai, and Chia-Yi Yu

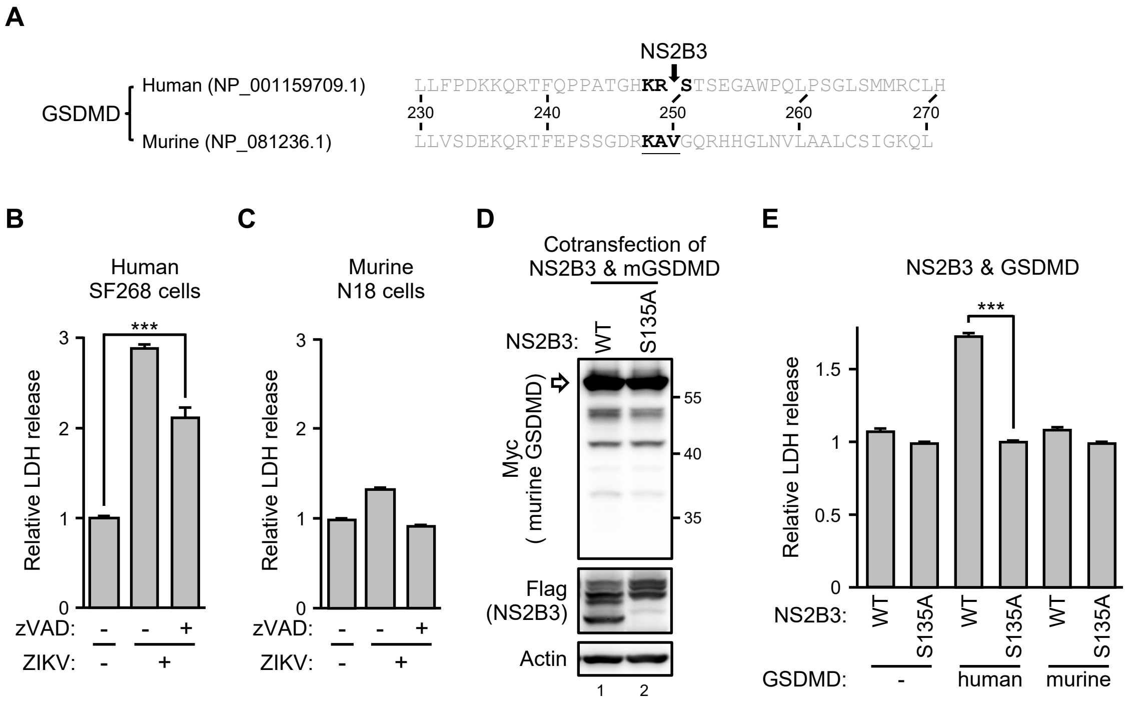

Figure S1. ZIKV inducing caspase-independent pyroptosis is species-dependent. (**A**) Corresponding amino acid sequences surrounding the ZIKV cleavage motif (KRS) of both human and murine GSDMD were aligned. (**B, C**) human glioblastoma SF268 cells (**B**) and murine neuroblastoma N18 cells (**C**) were infected with ZIKV in the absence (-) or presence (+) of zVAD (50 μM). (**D, E**) 293T/17 cells were cotransfected with ZIKV protease NS2B3 (WT, wild-type; S135A, the protease-dead mutant) and GSDMDs; lysates were examined by western blot analysis (**D**), and the supernatant was analyzed for LDH release (**E**). mGSDMD, murine GSDMD. Data are mean ± SD, *n* = 3 per group. ***p < 0.001.

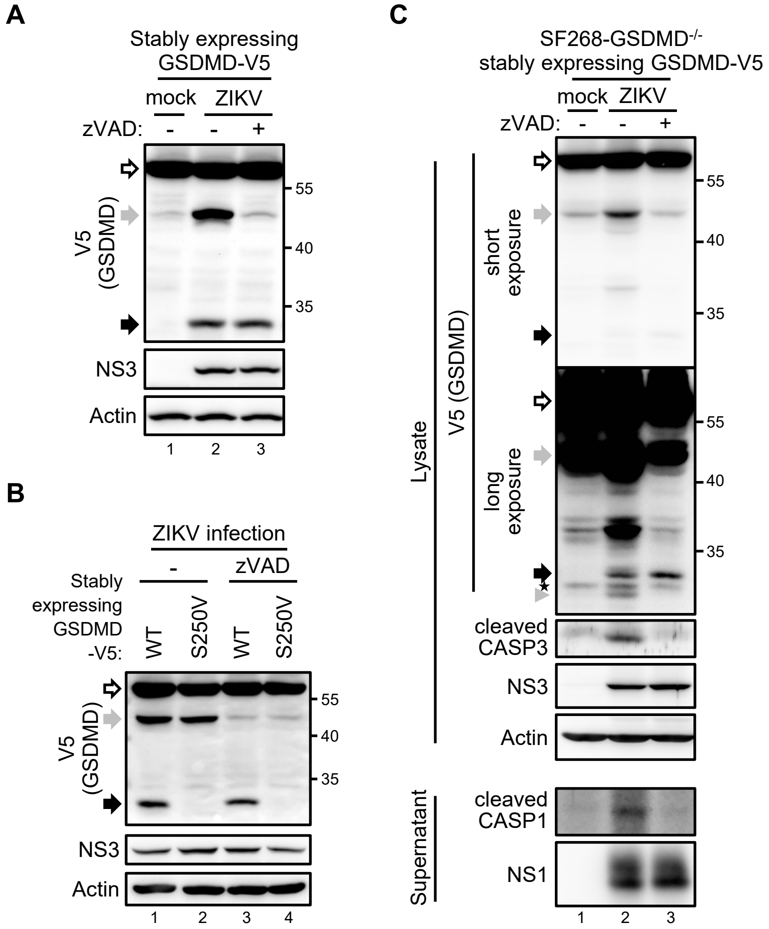

Figure S2. ZIKV-induced caspase-dependent and -independent GSDMD cleavage. (**A**) Mock- or ZIKV-infected Vero cells expressing full-length GSDMD-V5 in the absence (-) or presence (+) of zVAD (50 µM) were examined by western blot analysis. (**B**) Vero cells stably expressing GSDMD WT or its S250V mutant were infected with ZIKV in the absence (-) or presence (+) of zVAD (50 µM). (**C**) Mock- or ZIKV-infected SF268-GSDMD-/- cells expressing full-length GSDMD-V5 in the absence (-) or presence (+) of zVAD (50 µM) were examined by western blot analysis. CASP3, caspase-3; CASP1, caspase-1. White arrows, full-length; black arrows, ZIKV protease-cleaved; gray arrows, CASP3-cleaved; gray triangle, CASP1-cleaved; star, non-specific.

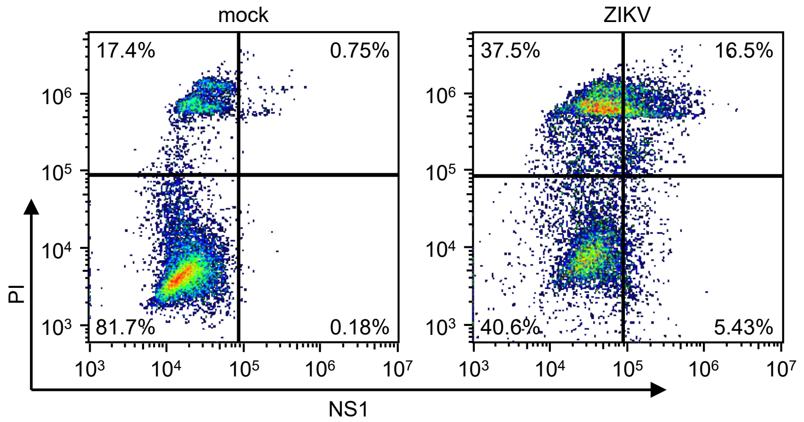

Figure S3. Uninfected cells could die from being exposed to a cytotoxic environment established by ZIKV-infected cells. SF268 cells were infected with ZIKV and live-stained for the cell surface NS1 and with PI, then analyzed by flow cytometry. NS1-negative, uninfected.

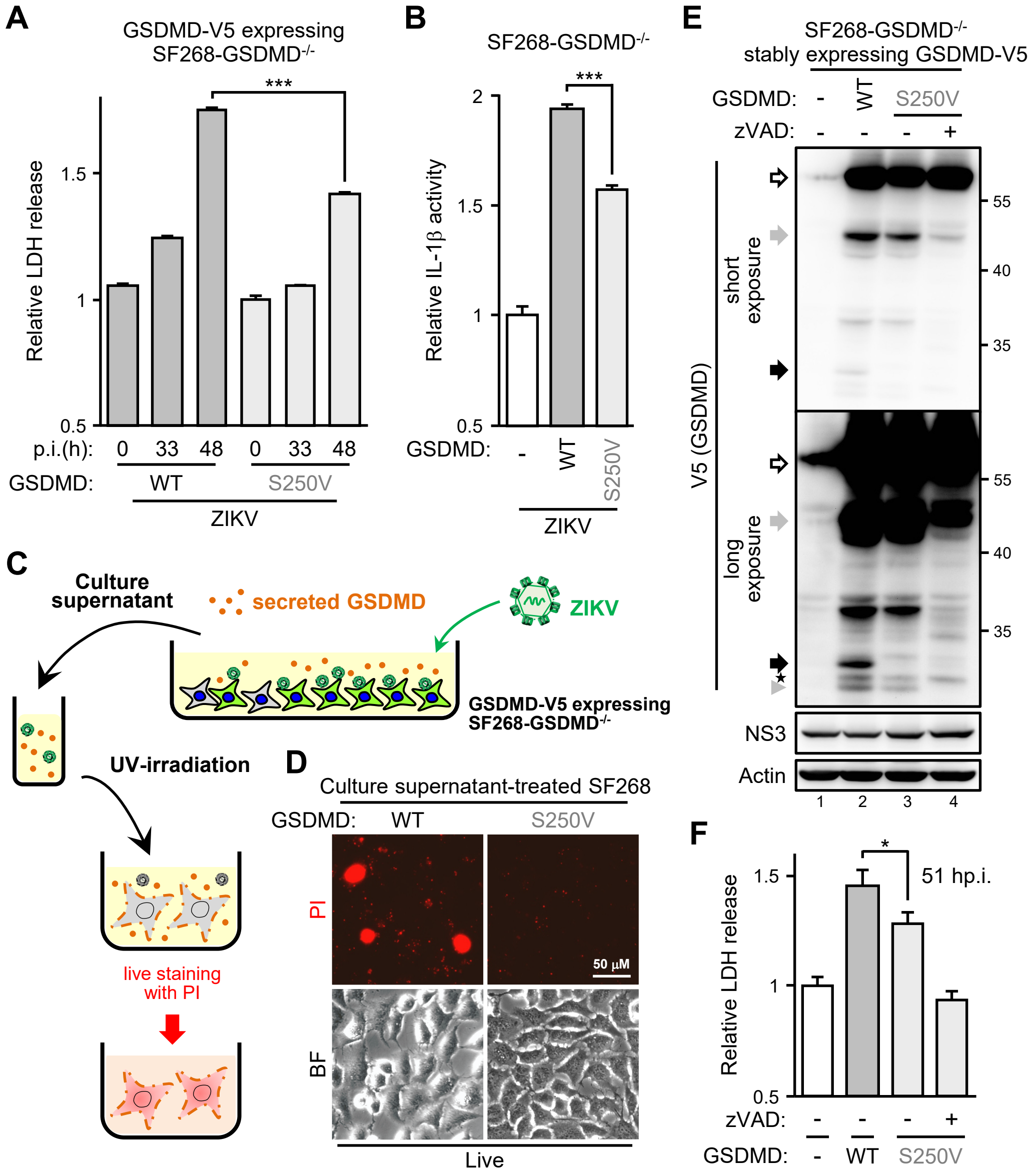

Figure S4. ZIKV-induced cytotoxicity was attenuated by the GSDMD S250V mutant. (**A**, **B**) SF268-GSDMD-/- cells stably expressing the V5-tagged GSDMD were infected with ZIKV as indicated. The supernatant was measured for LDH release (**A**) and IL-1β secretion (**B**). p.i., postinfection. (**C**, **D**) The culture supernatant from ZIKV-infected SF268-GSDMD-/- cells expressing the indicated GSDMD was UV-inactivated and used as the conditioned media incubating the naïve SF268 cells. Red, PI live-staining; BF, bright-field. (**E**, **F**) SF268-GSDMD-/- cells expressing the indicated GSDMD-V5 were infected with ZIKV in the absence (-) or presence (+) of zVAD (50 μM). The indicated protein levels were examined by western blot (**E**), and the supernatant was analyzed for LDH release (**F**). White arrows, full-length; black arrows, ZIKV protease-cleaved; gray arrows, CASP3-cleaved; gray triangle, CASP1-cleaved; star, non-specific. Data are mean ± SD, n = 3 per group. *$p < 0.05$, and ***$p < 0.001$.

A

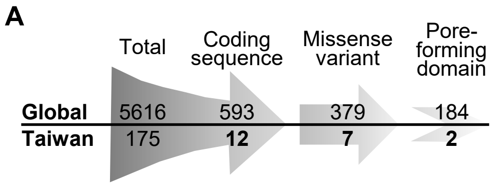

C

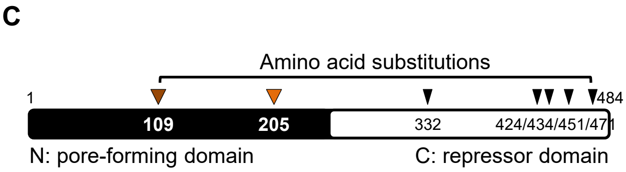

B

	SNP ID (rs#)	Frequency	Amino acid substitution
Pore forming domain	rs200806004	G:0.997026 A:0.002974	A109T
	rs11551200	C:0.999669 T:0.000331	R151@
	rs11551202	G:0.916888 A:0.083112	L164@
	rs112103340	G:0.999326 A:0.000674	T173@
	rs149736517	C:0.977657 T:0.022343	T205M
Repressor domain	rs147352021	C:0.999660 T:0.000340	A332V
	rs11551198	G:0.991421 A:0.008579	P341@
	rs372679903	G:0.999615 A:0.000385	T384@
	rs201141849	C:0.993991 T:0.006009	P424S
	rs200540390	G:0.986433 A:0.013567	E434K
	rs143727728	G:0.984472 A:0.015528	E451K
	rs11551199	G:0.999670 A:0.000330	A471T

@ synonymous substitution

D

	SNP ID (rs#)	rs138749323	rs140608348
	Missense substitution	R249H	F240L
GSDMD SNP frequency from multiple databases	GO-ESP	A=0.00447 (58/12966)	C=0.00015 (2/12990)
	PAGE_ STUDY	A=0.00766 (593/77390)	C=0.00005 (4/77578)
	ExAC	A=0.00243 (178/73334)	C=0.000010 (1/103382)
	ALFA	A=0.000655 (72/109840)	C=0.00000 (0/78940)
	1000G	A=0.0064 (32/5008)	-
	GnomAD	A=0.004714 (661/140234)	-

Figure S5. Representative GSDMD SNPs related to this study. (**A**) The number of GSDMD SNPs in the Taiwan BioBank and the global NCBI 1000 Genomes Project data (dbSNP) by the indicated selection strategy. (**B**) The 12 GSDMD SNPs residing within the coding sequence. (**C**) Schematic illustrations of human GSDMDs with the functional domain and the SNPs with amino acid substitution mentioned. (**D**) Frequency (%) of two GSDMD variants, R249H (SNP rs138749323) and F240L (SNP rs140608348), in the human population.

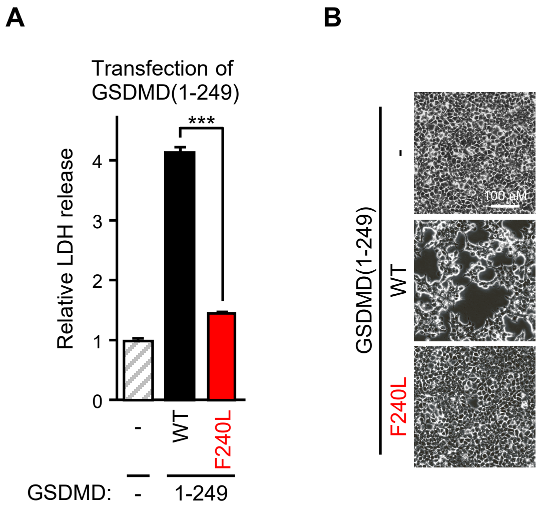

Figure S6. The cleaved GSDMD product with defective oligomerization lost its cytotoxicity. 293T/17 cells were transfected with GSDMD(1-249). The supernatant was collected for LDH assay (**A**). Data are mean ± SD (*n* = 3 per group). ***p < 0.001. Phase-contrast photography was shown in (**B**).

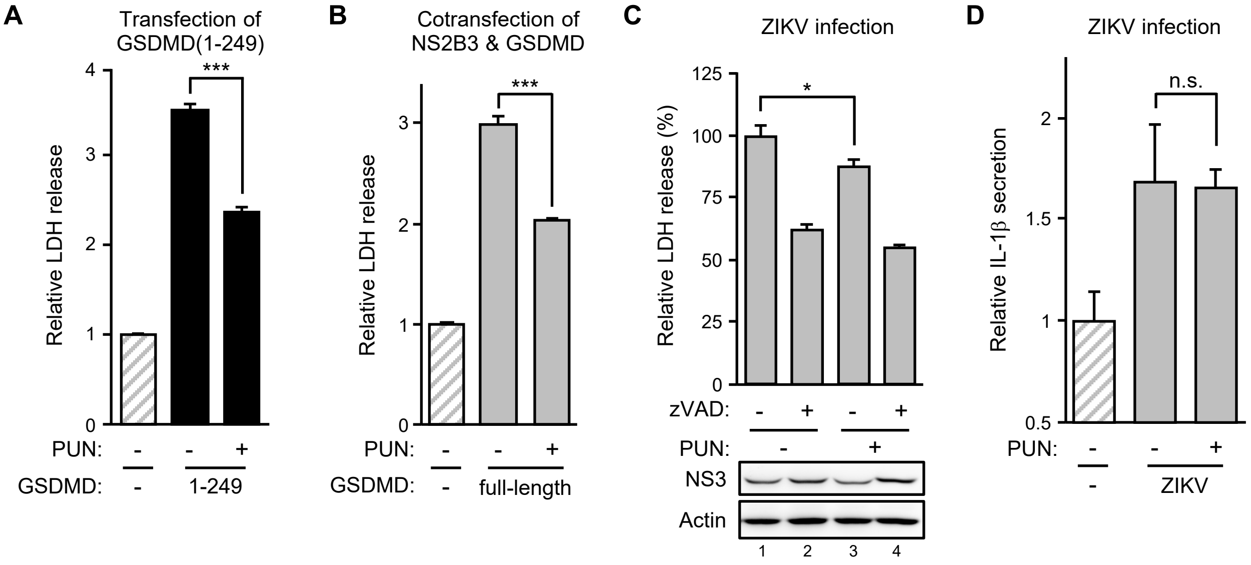

Figure S7. Punicalagin attenuates ZIKV-induced cytotoxicity. (**A**) 293T/17 cells were transfected with GSDMD(1-249) in the absence (-) or presence (+) of punicalagin (PUN, 10 μM). Released LDH was analyzed. (**B**) 293T/17 cells were cotransfected with full-length GSDMD and NS2B3 in the absence (-) or presence (+) of PUN (10 μM). Released LDH was analyzed. (**C, D**) SF268 cells were infected with ZIKV in the absence (-) or presence (+) of PUN (10 μM) or zVAD (50 μM). Released LDH (upper panel) and cell lysates (lower panel) were analyzed (**C**). IL-1β secretion (**D**) was determined. Data are mean ± SD, $n = 3$ per group. *$p < 0.05$, ***$p < 0.001$, and n.s., not significant.

Supplemental Methods and Materials

Inhibitor

Punicalagin (PUN) (P0023) was from Sigma-Aldrich (Saint Louis, MO, USA).

Cell line

Murine neuroblastoma N18 cells[1] were grown in RPMI (SH30027.01, HyClone, Logan, UT, USA) containing 5% FBS.

Plasmids

The GSDMD(1-249/F240L) was obtained by single-primer PCR mutagenesis with GSDMD(1-249)-V5 used as a template and the primer 5′ CGGATAAGAAGCAGAGGACCCTGCAGCCACCCGCGACA-3′. Murine GSDMD (MR207809) was from OriGene Technologies (Rockville, MD, USA).

Antibodies

The primary antibodies against ZIKV NS1 (YH0023) and Caspase-1 (#3866) were from Yao-Hong Biotechnology (Taiwan) and Cell Signaling Technology (Beverly, MA, USA), respectively.
